# Supplementary material for: Transverse oscillating bubble enhanced laser-driven betatron X-ray radiation generation
Source: Sci Rep. 2022 Jun 27;12:10855. doi: 10.1038/s41598-022-14748-z (PMC9237036; doi:10.1038/s41598-022-14748-z)
Supplement: Supplementary file 2 — Supplementary Information 2. [file 41598_2022_14748_MOESM2_ESM.pdf]

# Supplemental Material

## Movie of Particle-in-Cell (PIC) simulation

The movie of the particle-in-cell (PIC) simulation shows the dynamics the Transverse Oscillating Bubble Enhanced Betatron Radiation (TOBER) regime of the double-peaked "M" plasma target.

## Effective detection efficiencies and limits to spectral detection range

The range of the X-ray spectrometer is limited by beamline transmission at low photon energies (see Figure S1) and the quantum efficiency (see Figure S2) of the X-ray CCD camera for high energies. From the figures below, an effective detection efficiency that includes the filter transmission, the transmission of the thin Al foils and the quantum efficiency of the CCD camera (see Figure S2) of 1% was used to determine the detection limits of the spectrometer of 2.7 keV and 30 keV.

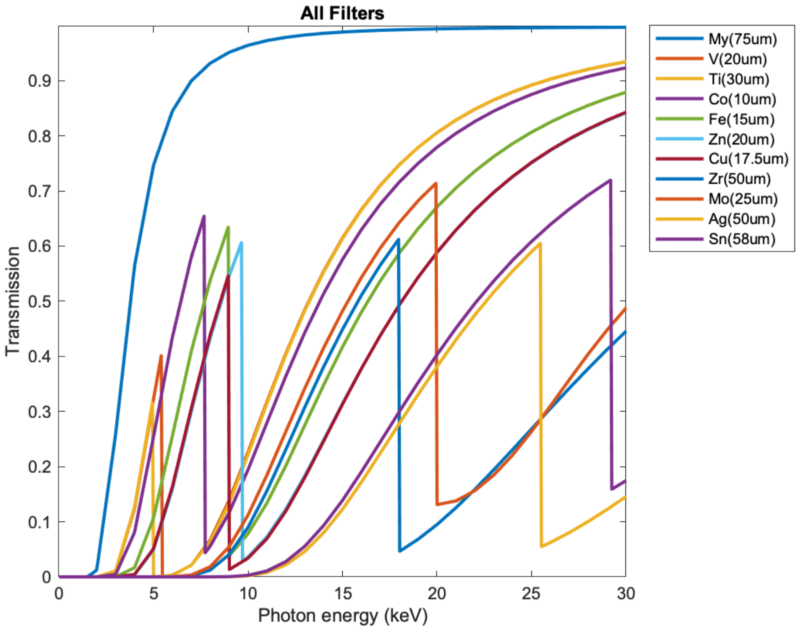


**Figure S1:** Transmission of the filters used in the experiment to determine the X-ray spectrum. Data from reference [S1].

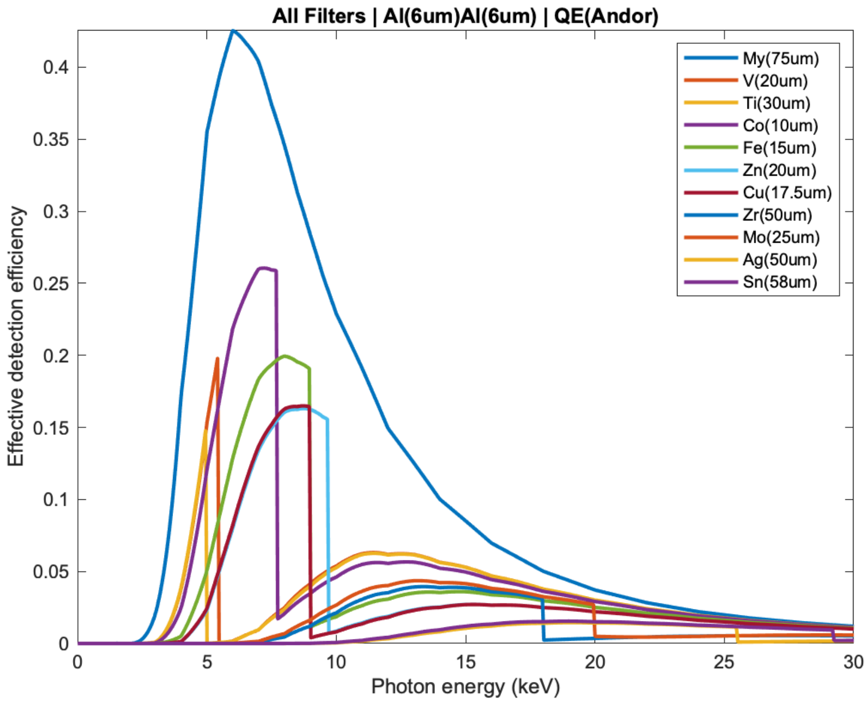


**Figure S2:** Effective detection efficiency for each filter used in the experiment including the transmission through the thin Al filters used in the beamline and including the quantum efficiency of the Andor X-ray CCD detector. An effective detection efficiency of 1% was used to determine the detection limits of the spectrometer of 2.7 keV and 30 keV.

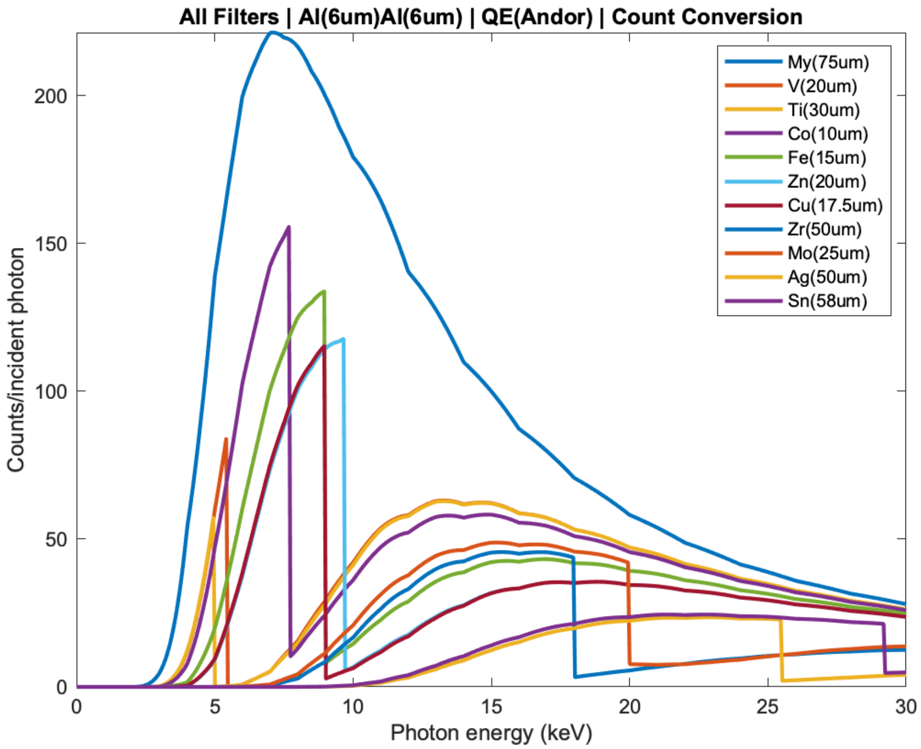


**Figure S3:** Effective analog-to-digital (ADU) counts of the camera for a single incident photon including the transmission through the filter, transmission through the thin Al filters used in the beamline and detector quantum efficiency.

## References


[S1] B.L. Henke, E.M. Gullikson, and J.C. Davis. *X-ray interactions: photoabsorption, scattering, transmission, and reflection at E=50-30000 eV, Z=1-92*, Atomic Data and Nuclear Data Tables Vol. **54** (no.2), 181-342 (July 1993).
